# TXNIP inhibition in the treatment of type 2 diabetes mellitus: design, synthesis, and biological evaluation of quinazoline derivatives

**DOI:** 10.1080/14756366.2023.2166937

**Published:** 2023-01-18

**Authors:** Aiyun Li, Li Guan, Wanzhen Su, Ning Zhao, Xuwen Song, Jin Wang, Xiaoxiao Tang, Weize Li, Xiangying Jiao

**Affiliations:** a Key Laboratory of Cellular Physiology (Shanxi Medical University), Ministry of Education, and the Department of Physiology, Shanxi Medical University, Taiyuan, PR China; b College of Pharmacy, Xi’an Medical University, Xi’an, PR China

**Keywords:** Quinazoline derivatives, TXNIP inhibition, oxidative stress and inflammation, pancreatic β cell apoptosis, T2DM

## Abstract

Thioredoxin interacting protein (TXNIP) is a potential drug target for type 2 diabetes mellitus (T2DM) treatment. A series of quinazoline derivatives were designed, synthesised, and evaluated to inhibit TXNIP expression and protect from palmitate (PA)-induced β cell injury. In vitro cell viability assay showed that compounds D-2 and C-1 could effectively protect β cell from PA-induced apoptosis, and subsequent results showed that these two compounds decreased TXNIP expression by accelerating its protein degradation. Mechanistically, compounds D-2 and C-1 reduced intracellular reactive oxygen species (ROS) production and modulated TXNIP-NLRP3 inflammasome signalling, and thus alleviating oxidative stress injury and inflammatory response under PA insult. Besides, these two compounds were predicted to possess better drug-likeness properties using SwissADME. The present study showed that compounds D-2 and C-1, especially compound D-2, were potent pancreatic β cell protective agents to inhibit TXNIP expression and might serve as promising lead candidates for the treatment of T2DM.

## Introduction

Diabetes mellitus is a chronic metabolic disease characterised by common outcome, hyperglycaemia, and is a major global problem spiralling out of control. The latest data, according to the International Diabetes Federation (IDF), showed that the number of diagnosed diabetes in adults reached 537 million in 2021 and which is predicted to rise to 643 million by 2030 and 783 million by 2045^1^. Type 2 diabetes mellitus (T2DM) accounts for more than 90% of all diabetes cases and is the leading cause of diabetes complications such as cardiovascular disease, diabetic nephropathy, diabetic neuropathy and diabetic retinopathy^2^. Although clinically used anti-diabetic drugs such as biguanides, sulfonylureas, thiazolidinediones and insulin have shown remarkable therapeutic effects, severe hypoglycaemia and weight gain are their undesirable side effects that limited their use^3^. Thus, developing new drugs for T2DM treatment meets great needs and prospects.

The pathogenesis of T2DM remains enigmatic and relative deficit in insulin secretion from pancreatic β cell emerges as the main culprit. Studies showed that loss of functional pancreatic β cell due to cell apoptosis or death is major event responsible for relative insulin deficiency in T2DM^4^^,^^5^. In this context, drugs preventing β cell failure is critical to revert the development of T2DM. Nowadays, the prevalence of obesity influenced by lifestyle factors is increasing and it is well established that obesity is a vital risk for T2DM^6^^,^^7^. Obesity is associated with elevated circulating free fatty acids (FFAs) which have a clear role in progressive pancreatic β cell dysfunction, impairing the ability of β cell to compensate for the prevailing insulin resistance and thus contributing to T2DM pathogenesis^8^. Among various saturated fatty acids, palmitic acid has the higher hazard ratio for pancreatic β cell dysfunction or apoptosis, suggesting a potential role for FFAs in diabetes development^9–11^. Exposure of β cell to palmitate (PA) presents critical features of β cell failure in T2DM via inducing cell apoptosis. Therefore, successfully protecting β cell from PA-induced apoptosis may potentially prevent T2DM^12^.

Thioredoxin-interacting protein (TXNIP), an endogenous negative modulator of thioredoxin (TRX), plays a vital role in maintaining the redox balance in the cell^13^. TRX is a redox protein with antioxidant effects and the conserved Trp-Cys (32)-Gly-Pro-Cys (35) sequence is the key functional structure^14^. TXNIP inhibits TRX activity by binding to the two cysteine residues in TRX and induces oxidative stress^15^. TXNIP is a crucial regulator of glucose homeostasis and plays an essential role in T2DM^16^^,^^17^. Researches showed that TXNIP is highly induced by hyperglycaemia in β cell and TXNIP deficiency exerts an antidiabetic effect via regulating pancreatic β cell function^18^^,^^19^. Inflammation and oxidative stress are crucial factors in the development of T2DM. Interestingly, previous studies have shown that TXNIP is not only involved in exacerbating oxidative stress but also leads to an increased inflammatory response in an NLRP3 inflammasome-dependent manner^20^. FFAs are potent inducers of cytosolic and mitochondrial reactive oxygen species (ROS) production in various cell types, including pancreatic β cell, through different mechanisms^21^. Excess FFAs increased ROS and result in the dissociation of TXNIP from TRX, then TXNIP bind to NLRP3 and subsequent activation of NLRP3 inflammasome^20^^,^^22^. PA-induced oxidative stress and inflammation via activating TXNIP, and thus cause β cell dysfunction and apoptosis. Therefore, inhibition of TXNIP could reduce oxidative stress and inflammatory injury of pancreatic β cell in T2DM, especially in the context of FFAs overload.

**Scheme 1.**
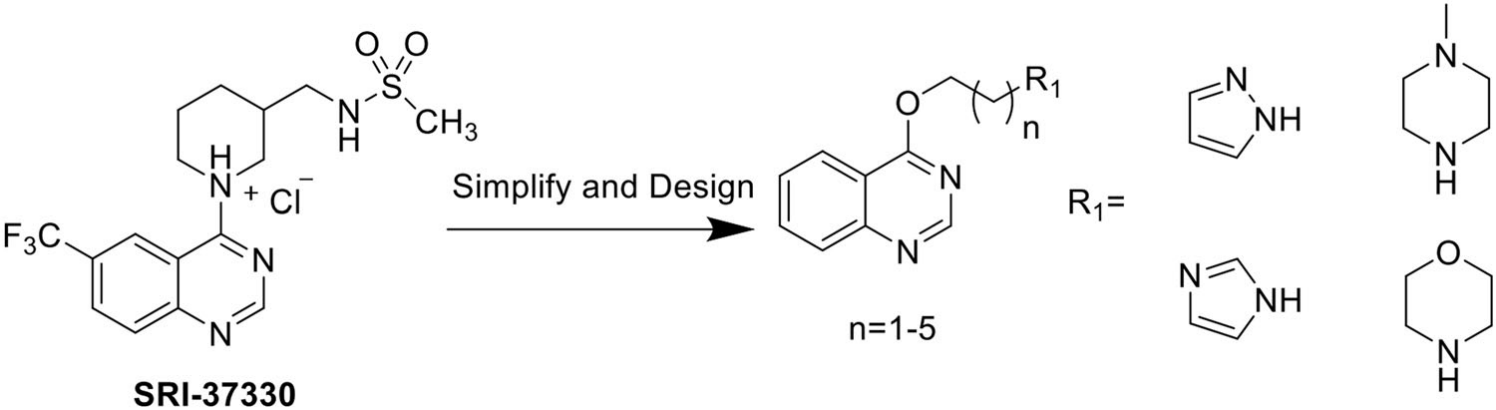
The design of target compounds.

The development of drugs to inhibit TXNIP expression could be an attractive approach for T2DM treatment. Recently, a compound named SRI-37330 was reported to inhibit TXNIP^23^. SRI-37330 is a quinazoline sulphonamide derivative; in addition to the quinazoline structure, a nitrogen-containing heterocycle is found in the molecule. Based on structural analysis and simplified compounds, we retained the quinazoline ring and nitrogen-containing heterocycle, and aimed to enrich compound libraries that could inhibit TXNIP. Here, 20 compounds were designed by combining unsubstituted quinazoline with different nitrogen-containing heterocycles through carbon chains of different lengths (Scheme 1). Subsequent *in vitro* pharmacological experiments and *in silico* physiochemical and pharmacokinetics prediction demonstrated that the compounds D-2 and C-1, especially the compound D-2, had better pancreatic β cell protective activity via inhibition of TXNIP expression and would be promising lead candidates for the treatment of T2DM.

## Materials and methods

### Chemistry

All chemicals were purchased from Aladdin Shanghai Biochemical Technology Co., Ltd. (China). Precoated silica gel GF254 (Qingdao Haiyang Chemical Plant, Qingdao, China) plate was used to monitor the reaction progress.

^1^H and ^13^C NMR spectra were measured on a Bruker 400 MHz spectrometer at 298 T and referenced to TMS. Chemical shifts were reported in ppm and splitting patterns were marked as s (singlet), d (doublet), t (triplet), m (multiplet).

Mass spectra were obtained on an MS Agilent 1100 Series LC/MSD Trap mass spectrometer (ESI-MS). Silica gel (200–300 mesh; Qingdao Marine Chemical Inc.) was used for column chromatography.

#### Synthetic intermediates

Dibromoalkanes with different length carbon chains and catalyst K_2_CO_3_ (4.17 g, 30.17 mmol) were dissolved in 30 mL DMF, then 4-hydroxyquinazoline (3.00 g, 20.12 mmol) was then dissolved in 10 mL of DMF and added dropwise at room temperature. After 0.5 h, the reaction was complete. The mixture was washed with 50 mL of water three times, and the organic layer was dried with anhydrous Na_2_SO_4_ and concentrated under reduced pressure to obtain the reaction product. The product was purified by column chromatography (petroleum ether: acetone = 7:1) to give the compound as a white crystal.

##### 4-(2-Bromoethoxy)quinazoline (A)

White crystal, 58.5% Yield; mp 99–100 °C; ^1^H-NMR (400 MHz, CDCl_3_) *δ* 8.31 (1H, dd, *J* = 1.2 Hz, 8.0 Hz, H-8), 8.10 (1H, s, H-2), 7.79 (1H, td, *J* = 1.4 Hz, 7.5 Hz, H-6), 7.74 (1H, d, *J* = 7.2 Hz, H-5), 7.53 (1H, dt, *J* = 1.2 Hz, 7.5 Hz, H-7), 4.39 (2H, t, *J* = 5.8 Hz, OCH_2_–), 3.78 (2H, t, *J* = 5.8 Hz, CH_2_Br); ^13^C-NMR (100 MHz, CDCl_3_) *δ* 161.0, 148.0, 146.6, 134.6, 127.6, 126.7, 121.9, 49.0, 29.6. ESI-MS *m/z*: 252.9 [M + H]^+^.

##### 4-(3-Bromopropoxy)quinazoline (B)

White crystal, 61.4% Yield; mp 115–116 °C. ^1^H-NMR (400 MHz, CDCl_3_) *δ* 8.31 (1H, dd, *J* = 1.0 Hz, 8.0 Hz, H-8), 8.11 (1H, s, H-2), 7.78 (1H, td, *J* = 1.5 Hz, 7.5 Hz, H-6), 7.72 (1H, dd, *J* = 0.8 Hz, 8.1 Hz, H-5), 7.52 (1H, td, *J* = 2.1 Hz, 7.5 Hz, H-7), 4.19 (2H, t, *J* = 6.7 Hz, OCH_2_–), 3.45 (2H, t, *J* = 6.1 Hz, CH_2_Br), 2.40 (2H, m, –CH_2_–); ^13^C-NMR (100 MHz, CDCl_3_) *δ* 161.2, 148.1, 146.6, 134.4, 127.6, 127.5, 126.6, 122.0, 45.6, 31.1, 29.9. ESI-MS *m/z*: 266.9 [M + H]^+^.

##### 4-(4-Bromobutoxy)quinazoline (C)

White crystal, 65.5% Yield; mp 75–77 °C. ^1^H-NMR (400 MHz, CDCl_3_) *δ* 8.32 (1H, dd, *J* = 1.2 Hz, 8.0 Hz, H-8), 8.04 (1H, s, H-2), 7.77 (1H, td, *J* = 1.4 Hz, 7.5 Hz, H-6), 7.72 (1H, d, *J* = 7.3 Hz, H-5), 7.52 (1H, td, *J* = 1.3 Hz, 7.6 Hz, H-7), 4.05 (2H, t, *J* = 6.8 Hz, OCH_2_–), 3.45 (2H, t, *J* = 6.3 Hz, CH_2_Br), 1.97 (4H, t, *J* = 3.6 Hz, –CH_2_CH_2_–); ^13^C-NMR (100 MHz, CDCl_3_) *δ* 161.0, 147.6, 146.5, 134.4, 127.5, 127.2, 126.7, 121.9, 46.1, 32.7, 29.6, 28.1. ESI-MS *m/z*: 281.0 [M + H]^+^.

##### (5-bromopentyl)oxy)quinazoline (D)

White crystal, 52.0% Yield; mp 55–56 °C. ^1^H-NMR (400 MHz, CDCl_3_) *δ* 8.31 (1H, dd, *J* = 1.1 Hz, 8.0 Hz, H-8), 8.04 (1H, s, H-2), 7.77 (1H, td, *J* = 1.4 Hz, 7.5 Hz, H-6), 7.71 (1H, d, *J* = 7.3 Hz, H-5), 7.52 (1H, td, *J* = 1.2 Hz, 7.5 Hz, H-7), 4.05 (2H, t, *J* = 6.8 Hz, OCH_2_–), 3.45 (2H, t, *J* = 6.3 Hz, CH_2_Br), 1.89 (6H, m, –CH_2_CH_2_CH_2_–); ^13^C-NMR (100 MHz, CDCl_3_) *δ* 161.1, 148.1, 146.5, 134.2, 127.4, 127.3, 126.7, 122.2, 66.9, 58.7, 53.7, 47.0, 29.3; ESI-MS *m/z*: 294.9 [M + H]^+^.

##### 4-((6-Bromohexyl)oxy)quinazoline (E)

White crystal, 48.8% Yield; mp 67–68 °C. ^1^H-NMR (400 MHz, CDCl_3_) *δ* 8.32 (1H, dd, *J* = 1.0 Hz, 8.0 Hz, H-8), 8.03 (1H, s, H-2), 7.76 (1H, td, *J* = 1.5 Hz, 7.5 Hz, H-6), 7.71 (1H, dd, *J* = 0.8 Hz, 8.0 Hz, H-5), 7.52 (1H, td, *J* = 1.3 Hz, 7.4 Hz, H-7), 4.01 (2H, t, *J* = 7.3 Hz, OCH_2_–), 3.40 (2H, t, *J* = 6.9 Hz, CH_2_Br), 1.85 (4H, m, –CH_2_CH_2_–), 1.47 (4H, m, –CH_2_CH_2_–); ^13^C-NMR (100 MHz, CDCl_3_) *δ* 161.0, 147.9, 146.6, 134.2, 127.3, 126.7, 122.1, 46.9, 33.7, 32.5, 29.2, 27.7, 25.8; ESI-MS *m/z*: 309.1 [M + H]^+^.

#### General procedure for preparation of 20 quinazoline derivatives (A-1∼E-4)

Dissolve the intermediate and nitrogen-containing heterocycle in 10 mL of acetonitrile; catalyst K_2_CO_3_ or Na_2_CO_3_ was added and reacted at 80 °C for 40 h. During the reaction, the GF254 silica gel plate was continuously used for monitoring. Water (30 mL) and ethyl acetate (3 × 30 mL) were added for extraction, and the organic layer was dried over anhydrous Na_2_SO_4_ and concentrated under reduced pressure.

##### 4-(2-(1H-pyrazol-1yl)ethoxy) quinazoline (A-1)

White crystal, 84.3% yield; mp 117–119 °C. ^1^H-NMR (400 MHz, CDCl_3_) *δ* 8.15 (1H, d, *J* = 7.6 Hz, H-8), 8.08 (1H, s, H-2), 7.70 (1H, t, *J* = 7.6 Hz, H-6), 7.60 (1H, s, H-5), 7.58 (1H, d, *J* = 5.2 Hz, pyrazole), 7.42 (1H, t, *J* = 7.6 Hz, H-7), 6.32 (1H, s, pyrazol), 4.14 (2H, t, *J* = 4.7 Hz, OCH_2_–), 3.40 (2H, t, *J* = 4.7 Hz, CH_2_N–); ^13^C-NMR (100 MHz, CDCl_3_) *δ* 161.4, 147.6, 147.2, 139.7, 134.4, 127.3, 127.0, 126.5, 122.1, 121.7, 105.1, 60.2, 49.8. ESI-MS *m/z*: 242.1 [M + H]^+^.

##### 4-(2-(4-Methylpiperazin-1-yl)ethoxy) quinazoline (A-2)

Yellow oil; 81.8% yield; ^1^H-NMR (400 MHz, CDCl_3_) *δ* 8.31 (1H, dd, *J* = 1.0 Hz, 8.0 Hz, H-8), 8.07 (1H, s, H-2), 7.76 (1H, td, *J* = 1.4 Hz, 7.5 Hz, H-6), 7.71 (1H, d, *J* = 7.3 Hz, H-5), 7.50 (1H, td, *J* = 1.3 Hz, 7.4 Hz, H-7), 4.09 (2H, t, *J* = 5.9 Hz, OCH_2_–), 2.72 (2H, t, *J* = 6.0 Hz, CH_2_N–), 2.56 (4H, s, NCH_2_CH_2_N), 2.44 (4H, s, NCH_2_CH_2_N), 2.27 (3H, s, –CH_3_); ^13^C-NMR (100 MHz, CDCl_3_) *δ* 161.4, 148.2, 147.2, 134.2, 127.4, 127.1, 126.7, 122.1, 56.5, 55.0, 53.2, 46.0, 43.7; ESI-MS *m/z*: 273.1 [M + H]^+^.

##### 4-(2-(1H-imidazol-1-yl)ethoxy) quinazoline (A-3)

White crystal, 50.4% yield, mp 140–141 °C. ^1^H-NMR (400 MHz, CDCl_3_) *δ* 8.32 (1H, dd, *J* = 1.3 Hz, 8.0 Hz, H-8), 7.79 (1H, td, *J* = 1.4 Hz, 7.6 Hz, H-6), 7.69 (1H, d, *J* = 8.1 Hz, H-5), 7.55 (1H, t, *J* = 8.0 Hz, H-7), 7.46 (1H, s, H-8), 7.39 (1H, s, imidazol), 7.09 (1H, s, imidazol), 6.87 (1H, s, imidazol) 4.42 (2H, t, *J* = 5.5 Hz, OCH_2_–), 4.3 (2H, t, *J* = 5.4 Hz, CH_2_N–); ^13^C-NMR (100 MHz, CDCl_3_) *δ* 161.1, 148.0, 145.5, 137.2, 135.0, 134.8, 130.6, 127.8, 126.6, 121.6, 118.7, 48.5, 44.9; ESI-MS *m/z*: 241.1 [M + H]^+^.

##### (4-(2-(quinazolin-4-yloxy)ethyl)morpholine (A-4)

White crystal, 85.8% yield, mp 74–76 °C. ^1^H-NMR (400 MHz, CDCl_3_) *δ* 8.31 (1H, dd, *J* = 1.3 Hz, 8.0 Hz, H-8), 8.07 (1H, s, H-2), 7.77 (1H, td, *J* = 1.4 Hz, 7.5 Hz, H-6), 7.71 (1H, d, *J* = 7.8 Hz, H-5), 7.51 (1H, td, *J* = 1.2 Hz, 7.4 Hz, H-7), 4.10 (2H, t, *J* = 5.9 Hz, OCH_2_–), 3.68 (4H, t, *J* = 4.5 Hz, NCH_2_CH_2_O), 2.72 (2H, t, *J* = 6.0 Hz, CH_2_N–), 2.52 (4H, d, *J* = 4.5 Hz, NCH_2_CH_2_O); ^13^C-NMR (100 MHz, CDCl_3_) *δ* 161.1, 148.1, 147.1, 145.5, 134.2, 127.4, 126.7, 122.0, 66.9, 57.0, 53.7, 43.4; ESI-MS *m/z*: 260.1 [M + H]^+^.

##### 4-(3-(1H-pyrazol-1-yl)propoxy) quinazoline (B-1)

Yellow oil, 91.1% yield; ^1^H-NMR (400 MHz, CDCl_3_) *δ* 8.31(1H, td, *J* = 1.1 Hz, 6.9 Hz, H-8), 8.10 (1H, d, *J* = 8.1 Hz, H-2), 7.78 (1H, m, H-6), 7.72 (1H, t, *J* = 5.3 Hz, H-5), 7.63 (1H, d, *J* = 1.9 Hz, pyrazole), 7.52 (1H, td, *J* = 1.3 Hz, 7.4 Hz, H-7), 6.27 (1H, t, *J* = 2.0 Hz, pyrazol), 2.41 (2H, m, OCH_2_–), 2.04 (2H, m, CH_2_N–); ^13^C-NMR (100 MHz, CDCl_3_) *δ* 161.3, 148.1, 146.8, 139.7, 134.4, 129.4, 127.5, 126.7, 122.0, 121.8, 105.8, 58.1, 48.7, 32.0; ESI-MS *m/z*: 255.1 [M + H]^+^.

##### 4-(3-(4-Methylpiperazin-1-yl)propoxy) quinazoline (B-2)

Yellow oil, 81.8% yeild; ^1^H-NMR (400 MHz, CDCl_3_) *δ* 8.31 (1H, dd, *J* = 1.0 Hz, 8.0 Hz, H-8), 8.14 (1H, s, H-2), 7.76 (1H, td, *J* = 1.5 Hz, 7.6 Hz, H-6), 7.71 (1H, dd, *J* = 0.8 Hz, 8.1 Hz, H-5), 7.51 (1H, td, *J* = 1.3 Hz, 7.5 Hz, H-7), 4.09 (2H, t, *J* = 6.6 Hz, OCH_2_–), 2.44 (4H, s, NCH_2_CH_2_N), 2.38 (2H, t, *J* = 6.6 Hz, CH_2_N–), 2.27 (3H, s, –CH_3_), 2.03 (4H, m, NCH_2_CH_2_N); ^13^C-NMR (100 MHz, CDCl_3_) *δ* 161.1, 148.1, 147.3, 134.1, 127.3, 127.1, 126.5, 122.1, 55.0, 54.1, 52.6, 45.8, 45.0, 25.1; ESI-MS *m/z*: 287.1 [M + H]^+^.

##### 4-(3-(1H-imidazol-1-yl)propoxy) quinazoline (B-3)

Yellow oil, 63.1% yeild; ^1^H-NMR (400 MHz, CDCl_3_) *δ* 8.31(1H, dd, *J* = 1.4 Hz, 7.4 Hz, H-8), 7.95 (1H, s, H-2), 7.79 (1H, td, *J* = 1.5 Hz, 7.6 Hz, H-6), 7.72 (1H, d, *J* = 7.7 Hz, H-5), 7.58 (1H, s, imidazol)), 7.54 (1H, td, *J* = 1.2 Hz, 7.5 Hz, H-7), 7.12 (1H, s, imidazol), 7.00 (1H, s, imidazol), 4.09 (2H, t, *J* = 7.0 Hz, OCH_2_–), 4.01 (2H, t, *J* = 7.2 Hz, CH_2_N–), 2.05 (1H, m, –CH_2_–), 2.26 (1H, t, *J* = 7.2 Hz, –CH_2_–); ^13^C-NMR (100 MHz, CDCl_3_) *δ* 161.2, 148.0, 146.8, 146.0, 137.0, 135.1, 129.7, 127.7, 126.6, 121.9, 118.8, 58.0, 44.3, 30.6; ESI-MS *m/z*: 255.1 [M + H]^+^.

##### 4-(3-(Quinazolin-4-yloxy)propyl) morpholine (B-4)

Yellow oil, 98.1% yeild; ^1^H-NMR (400 MHz, CDCl_3_) *δ* 8.31 (1H, d, *J* = 8.0 Hz, H-8), 8.13 (1H, s, H-2), 7.76 (1H, td, *J* = 2.1 Hz, 6.9 Hz, H-6), 7.70 (1H, d, *J* = 8.2 Hz, H-5), 7.51 (1H, t, *J* = 7.7 Hz, H-7), 4.10 (2H, t, *J* = 6.6 Hz, OCH_2_–), 3.67 (4H, s, NCH_2_CH_2_O), 2.41 (4H, d, J = 2.4 Hz, NCH_2_CH_2_O), 2.39 (2H, t, J = 6.5 Hz, CH_2_N-); ^13^C-NMR (100 MHz, CDCl_3_) *δ* 161.1, 148.0, 147.2, 134.1, 127.3, 127.1, 126.5, 122.0, 66.8, 54.8, 53.5, 45.1, 24.8; ESI-MS *m/z*: 274.1 [M + H]^+^.

##### (4-(4-(1H-pyrazol-1-yl)butoxy) quinazoline (C-1)

White crystal, 82.9% yield; mp 106–108 °C. ^1^H-NMR (400 MHz, CDCl_3_) *δ* 8.31 (1H, td, *J* = 1.2 Hz, 7.9 Hz, H-8), 8.04 (1H, s, H-2), 7.77 (1H, td, *J* = 1.5 Hz, 7.5 Hz, H-6), 7.71 (1H, dd, *J* = 0.6 Hz, 8.0 Hz, H-5), 7.51 (1H, t, *J* = 1.3 Hz, 7.4 Hz, H-7), 6.27 (1H, t, *J* = 2.0 Hz, pyrazol), 4.03 (2H, t, *J* = 7.3 Hz, OCH_2_–), 2.04 (2H, m, CH_2_N–), 1.25 (4H, t, *J* = 3.6 Hz, –CH_2_CH_2_–); ^13^C-NMR (100 MHz, CDCl_3_) *δ* 161.2, 147.9, 146.4, 139.5, 134.3, 129.3, 127.5, 127.3, 126.6, 122.1, 105.4, 51.3, 46.1, 27.4, 26.5; ESI-MS *m/z*: 269.1 [M + H]^+^.

##### 4-(4-(4-Methylpiperazin-1-yl)butoxy) quinazoline (C-2)

Yellow oil, 61.1% yeild; ^1^H-NMR (400 MHz, CDCl_3_) *δ* 8.31 (1H, dd, *J* = 1.0 Hz, 7.9 Hz, H-8), 8.04 (1H, s, H-2), 7.76 (1H, td, *J* = 1.5 Hz, 7.5 Hz, H-6), 7.71 (1H, dd, *J* = 0.8 Hz, 7.6 Hz, H-5), 7.51 (1H, td, *J* = 1.3 Hz, 7.4 Hz, H-7), 4.03 (2H, t, *J* = 7.3 Hz, OCH_2_–), 2.45 (4H, s, NCH_2_CH_2_N), 2.39 (2H, t, *J* = 7.4 Hz, CH_2_N–), 2.28 (3H, s, –CH_3_), 1.83 (2H, m, –CH_2_CH_2_–), 1.58 (2H, m, –CH_2_CH_2_–), 1.25 (4H, s, NCH_2_CH_2_N); ^13^C-NMR (100 MHz, CDCl_3_) *δ* 161.1, 148.1, 147.3, 134.1, 127.3, 127.1, 126.5, 122.1, 55.0, 54.1, 52.6, 45.8, 45.0, 27.1, 25.1; ESI-MS *m/z*: 301.1 [M + H]^+^.

##### 4-(4-(1H-imidazol-1-yl)butoxy) quinazoline (C-3)

Yellow oil, 57.2% yeild; ^1^H-NMR (400 MHz, CDCl_3_) *δ* 8.30 (1H, dd, *J* = 0.8 Hz, 8.0 Hz, H-8), 8.00 (1H, s, H-2), 7.78 (1H, td, *J* = 1.4 Hz, 7.6 Hz, H-6), 7.72 (1H, d, *J* = 8.0 Hz, H-5), 7.69 (1H, s, imidazol), 7.53 (1H, t, *J* = 7.0 Hz, H-7), 7.50 (1H, s, imidazol), 7.10 (2H, s, CH_2_N–), 6.92 (1H, s, imidazol), 4.02 (2H, t, *J* = 7.0 Hz, OCH_2_–), 1.84 (4H, m, –CH_2_CH_2_–); ^13^C-NMR (100 MHz, CDCl_3_) *δ* 161.2, 148.0, 146.2, 137.0, 135.2, 134.3, 129.5, 127.5, 126.6, 121.9, 118.9, 46.4, 46.0, 29.7, 28.1; ESI-MS *m/z*: 269.1 [M + H]^+^.

##### (4-(4-(quinazolin-4-yloxy)butyl)morpholine (C-4)

White crystal, 85.8% yield; mp 81–82 °C. ^1^H-NMR (400 MHz, CDCl_3_) *δ* 8.31 (1H, dd, *J* = 1.0 Hz, 8.0 Hz, H-8), 8.04 (1H, s, H-2), 7.76 (1H, td, *J* = 1.5 Hz, 7.8 Hz, H-6), 7.71 (1H, d, *J* = 7.7 Hz, H-5), 7.51 (1H, td, *J* = 1.3 Hz, 7.2 Hz, H-7), 4.04 (2H, t, *J* = 6.6 Hz, OCH_2_–), 3.70 (4H, t, *J* = 4.6 Hz, NCH_2_CH_2_O), 2.42 (4H, t, NCH_2_CH_2_O), 2.38 (2H, t, *J* = 7.4 Hz, CH_2_N–), 1.85 (2H, m, –CH_2_–), 1.58 (2H, m, –CH_2_–); ^13^C-NMR (100 MHz, CDCl_3_) *δ* 161.1, 148.1, 146.5, 137.1, 134.2, 127.5, 126.7, 122.1, 66.9, 58.3, 53.7, 46.8, 27.4, 23.6; ESI-MS *m/z*: 288.1 [M + H]^+^.

##### 4-((5-(1H-pyrazol-1-yl) pentyl)oxy) quinazoline (D-1)

White crystal, 83.1% yield; mp 93–94 °C. ^1^H-NMR (400 MHz, CDCl_3_) *δ* 8.31 (1H, d, *J* = 8.0 Hz, H-8), 7.99 (1H, s, H-2), 7.76 (1H, td, *J* = 1.2 Hz, 7.6 Hz, H-6), 7.71 (1H, d, *J* = 7.8 Hz, H-5), 7.52 (1H, t, *J* = 7.9 Hz, H-7),7.49 (1H, s, pyrazol), 7.34 (1H, dd, *J* = 2.1 Hz, 8.4 Hz, pyrazol), 6.21 (1H, t, *J* = 2.1 Hz, pyrazol), 3.97 (2H, t, *J* = 7.3 Hz, OCH_2_–), 4.03 (2H, t, *J* = 7.3 Hz, OCH_2_–), 2.04 (2H, m, CH_2_N–), 1.82 (2H, m, –CH_2_–), 1.37 (2H, m, –CH_2_–), 1.26 (2H, m, –CH_2_–); ^13^C-NMR (100 MHz, CDCl_3_) *δ* 161.0, 147.8, 146.6, 134.3, 127.4, 127.3, 126.7, 122.1, 46.9, 33.3, 32.1, 28.5, 25.2; ESI-MS *m/z*: 283.1 [M + H]^+^.

##### 4-((5-(4-Methylpiperazin-1-yl) pentyl)oxy)quinazoline (D-2)

White crystal, 86.1% yield; mp 85–86 °C. ^1^H-NMR (400 MHz, CDCl_3_) *δ* 8.31 (1H, d, *J* = 8.0 Hz, H-8), 8.03 (1H, s, H-2), 7.76 (1H, td, *J* = 1.2 Hz, 7.5 Hz, H-6), 7.71 (1H, d, *J* = 8.1 Hz, H-5), 7.51 (1H, t, *J* = 6.9 Hz, H-7), 4.00 (2H, t, *J* = 7.4 Hz, OCH_2_–), 2.46 (4H, s, NCH_2_CH_2_N), 2.34 (2H, t, *J* = 7.6 Hz, CH_2_N–), 2.28 (3H, s, –CH_3_), 1.82 (2H, m, –CH_2_–), 1.56 (2H, m, –CH_2_–), 1.40 (2H, m, –CH_2_–); ^13^C-NMR (100 MHz, CDCl_3_) *δ* 161.1, 148.1, 146.5, 139.3, 129.0, 127.4, 127.3, 126.7, 122.1, 105.3, 58.2, 55.6, 51.8, 51.7, 46.8, 29.9, 28.8, 23.7; ESI-MS *m/z*: 315.1 [M + H]^+^.

##### 4-((5-(1H-imidazol-1-yl)pentyl)oxy) quinazoline (D-3)

Yellow oil, 46.6% yeild; ^1^H-NMR (400 MHz, CDCl_3_) *δ* 8.31 (1H, dd, *J* = 1.2 Hz, 8.0 Hz, H-8), 8.00 (1H, s, H-2), 7.77 (1H, td, *J* = 1.5 Hz, 7.5 Hz, H-6), 7.72 (1H, s, imidazol), 7.71 (1H, d, *J* = 2.9 Hz, H-5), 7.52 (1H, td, *J* = 1.2 Hz, 7.0 Hz, H-7), 7.50 (1H, s, imidazol), 6.92 (1H, s, imidazol), 3.97 (2H, m, OCH_2_–), 1.85 (4H, m, –CH_2_CH_2_–), 1.39 (2H, m, –CH_2_–); ^13^C-NMR (100 MHz, CDCl_3_) *δ* 161.1, 148.1, 146.5, 139.3, 129.0, 127.4, 126.7, 121.9, 122.1, 118.7, 105.3, 53.1, 47.0, 29.3, 26.4, 24.6; ESI-MS *m/z*: 283.1 [M + H]^+^.

##### 4-(5-(Quinazolin-4-yloxy)pentyl) morpholine (D-4)

White crystal, 65.4% yield; mp 81–82 °C. ^1^H-NMR (400 MHz, CDCl_3_) *δ* 8.31 (1H, dd, *J* = 1.0 Hz, 8.0 Hz, H-8), 8.03 (1H, s, H-2), 7.76 (1H, td, *J* = 1.5 Hz, 7.5 Hz, H-6), 7.71 (1H, d, *J* = 7.4 Hz, H-5), 7.51 (1H, td, *J* = 1.2 Hz, 7.5 Hz, H-7), 4.01 (2H, t, *J* = 7.3 Hz, OCH_2_–), 3.70 (4H, t, *J* = 4.6 Hz, NCH_2_CH_2_O), 2.41 (4H, t, *J* = 4.6 Hz, NCH_2_CH_2_O), 2.33 (2H, t, *J* = 7.4 Hz, CH_2_N–), 1.83 (2H, m, –CH_2_–), 1.55 (2H, m, –CH_2_–), 1.42 (2H, m, –CH_2_–); ^13^C-NMR (400 MHz, CDCl_3_) *δ* 161.2, 148.1, 146.3, 137.0, 134.3, 129.4, 127.5, 126.7, 122.0, 66.7, 54.8, 46.7, 46.3, 30.6, 28.8, 23.6. ESI-MS *m/z*: 302.2 [M + H]^+^.

##### 4-((6-(1H-pyrazol-1-yl)hexyl)oxy) quinazoline (E-1)

White crystal, 47.7% yield; mp 63–64 °C. ^1^H-NMR (400 MHz, CDCl_3_) *δ* 8.31 (1H, dd, *J* = 1.08 Hz,8.0 Hz, H-8), 8.00 (1H, s, H-2), 7.76 (1H, td, *J* = 1.4 Hz, 7.5 Hz, H-6), 7.71 (1H, d, *J* = 7.3 Hz, H-5), 7.5 (1H, td, *J* = 1.2 Hz, 7.5 Hz, H-7), 7.48 (1H, d, *J* = 1.5 Hz, pyrazol), 7.35 (1H, d, *J* = 2.0 Hz, pyrazol) 6.22 (1H, t, *J* = 2.0 Hz, pyrazol), 4.12 (2H, t, *J* = 7.0 Hz, OCH_2_–), 3.97 (2H, t, *J* = 7.3 Hz,CH_2_N–), 4.03 (2H, t, *J* = 7.3 Hz, OCH_2_–), 1.89 (2H, m, –CH_2_–), 1.87 (2H, m, –CH_2_–), 1.37 (4H, m, –CH_2_CH_2_–); ^13^C-NMR (100 MHz, CDCl_3_) *δ* 161.0, 148.1, 146.5, 139.1, 134.2, 128.9, 127.4, 127.2, 126.6, 122.1, 105.3, 51.9, 46.8, 30.2, 29.2, 26.1; ESI-MS *m/z*: 297.1 [M + H]^+^.

##### 4-((6-(4-Methylpiperazin-1-yl) hexyl)oxy)quinazoline (E-2)

White crystal, 72.0% yield; mp 47–48 °C. ^1^H-NMR (400 MHz, CDCl_3_) *δ* 8.31 (1H, d, *J* = 8.0 Hz, H-8), 8.03 (1H, s, H-2), 7.76 (1H, td, *J* = 1.5 Hz, 7.5 Hz, H-6), 7.71 (1H, d, *J* = 7.7 Hz, H-5), 7.51 (1H, t, *J* = 8.1 Hz, H-7), 4.00 (2H, t, *J* = 7.3 Hz, OCH_2_–), 2.4 (4H, s, NCH_2_CH_2_N), 2.34 (2H,d, *J* = 4.4 Hz, CH_2_N–), 2.28 (3H, s, –CH_3_), 1.81 (2H, m, –CH_2_–), 1.50 (2H, m, –CH_2_–), 1.38 (4H, m, –CH_2_CH_2_–); ^13^C-NMR (100 MHz, CDCl_3_) *δ* 161.1, 148.1, 146.6, 134.2, 127.4, 127.2, 126.7, 122.1, 105.3, 55.0, 54.1, 52.6, 51.9, 46.8, 30.2, 29.2, 26.1, 25.1; ESI-MS *m/z*: 329.2 [M + H]^+^.

##### 4-((6-(1H-imidazol-1-yl)hexyl)oxy) quinazoline (E-3)

Yellow oil, 48.1% yeild; ^1^H-NMR (400 MHz, CDCl_3_) *δ* 8.31 (1H, dd, *J* = 1.1 Hz, 8.0 Hz, H-8), 8.01 (1H, s, H-2), 7.77 (1H, td, *J* = 1.5 Hz, 7.5 Hz, H-6), 7.71 (1H, d, *J* = 7.2 Hz, H-5), 7.52 (1H, td, *J* = 1.2 Hz, 7.5 Hz, H-7), 7.46 (1H, s, imidazol), 7.04 (1H, s, imidazol), 6.89 (1H, s, imidazol), 3.99 (2H, t, *J* = 7.3 Hz, –CH_2_N), 3.99 (2H, t, *J* = 7.0 Hz, OCH_2_–), 1.79 (4H, m, –CH_2_CH_2_–), 1.38 (4H, m, –CH_2_CH_2_–); ^13^C-NMR (100 MHz, CDCl_3_) *δ* 161.1, 148.1, 146.6, 137.0, 135.2, 134.2, 127.4, 127.3, 126.7, 122.2, 118.9, 66.9, 58.9, 53.7, 47.0, 29.3, 27.0, 26.6, 26.3; ESI-MS *m/z*: 297.2 [M + H]^+^.

##### 4-(6-(Quinazolin-4-yl)oxy)hexyl) morpholine (E-4)

White crystal, 97.8% yield; mp 56–58 °C. ^1^H-NMR (400 MHz, CDCl_3_) *δ* 8.31 (1H, dd, *J* = 1.1 Hz, 8.1 Hz, H-8), 8.03 (1H, s, H-2), 7.76 (1H, td, *J* = 1.5 Hz, 7.5 Hz, H-6), 7.71 (1H, d, *J* = 7.7 Hz, H-5), 7.51 (1H, td, *J* = 1.3 Hz, 7.5 Hz, H-7), 4.00 (2H, t, *J* = 7.3 Hz, OCH_2_–), 3.70 (4H, t, *J* = 4.5 Hz, NCH_2_CH_2_O), 2.42 (4H, s, NCH_2_CH_2_O), 2.31 (2H, t, *J* = 7.8 Hz, CH_2_N–), 1.81 (2H, m, –CH_2_–), 1.50 (2H, m, –CH_2_–), 1.39 (4H, m, –CH_2_CH_2_–); ^13^C-NMR (100 MHz, CDCl_3_) *δ* 161.1, 148.0, 146.5, 137.0, 134.3, 129.2, 127.4, 122.1, 66.8, 63.2, 53.1, 46.9, 46.8, 30.8, 29.2, 26.1; ESI-MS *m/z*: 316.2 [M + H]^+^.

### Biological activity assay

#### Materials

Palmitate (PA) was from Solarbio science & technology Co., Ltd. (Beijing, China) and dissolved in ethanol, and then diluted with 50 mmol/L NaOH to prepare the stock solution. The stock solution was further diluted with DMEM containing 0.55% FFA-free BSA (Solarbio, Beijing, China) at the ratio of 1:100 before use. Cycloheximide and MG-132 were from MedChem Express (Shanghai, China).

#### Cell cultures

Min6 mouse pancreatic β cell were obtained from the ATCC cell bank. The cells were cultured in Dulbecco’s modified Eagle’s medium (DMEM, HyClone, USA) supplemented with 10% foetal bovine serum (FBS, Gibico, USA) and 1% Penicillin-Streptomycin Liquid (Solarbio, Beijing, China). Cell cultures were incubated in a humidified incubator containing 5% CO_2_ at 37 °C.

#### Cell counting kit-8 (CCK-8) assay

CCK-8 assay was performed to determine the cell viability under PA treatment and the protective activity of the tested compounds in PA-treated Min6 cells. In brief, Min6 cells were seeded in 96-well plates and cultured in DMEM overnight. Min6 cells were treated with tested compounds at indicated concentration and PA (300 μmol/L) for 24 h. After washing cells with PBS, fresh DMEM and CCK-8 reagents (Meilunbio, Dalian, China) were added and cells were incubated for 2 h in a 37 °C-incubator containing 5% CO_2_. Absorbance at 450 nm was read on a microplate reader (Molecular Devices, SpectraMax 190, USA).

#### RNA isolation and quantitative real-time PCR

Min6 cells were cultured into six-well plates with overnight culture. The cells were incubated with 10 μmol/L tested compounds and 300 μmol/L PA for 24 h. After washing with PBS, cells were lysed with TRIzol Reagent (Takara, Japan) to obtain total mRNA. cDNA was synthesised using PrimeScript RT Master Mix (TaKaRa, Japan) according to the manufacturer’ instructions. The quantity of IL-1β and IL-6 mRNA was detected by quantitative real-time PCR (qRT-PCR) using the TB Green Premix Ex Taq (TaKaRa, Japan). The relative values of mRNA were normalised with the levels of β-actin mRNA. The primers used were shown in Table 1.

**Table 1.** Primer sequences for real-time PCR assays.

Gene	Forward primer (5′-3′)	Reverse primer (5′-3′)
IL-1β	CGCAGCAGCACATCA ACAAGAGC	TGTCCTCATCCTGGAAGGTCCACG
IL-6	CCACTTCACAAGTCGGAGGCTTA	TGCAAGTGCATCATCGTTGTTC
β-actin	CACTATTGGCAACGAGCGGTTCCG	ACGGATGTCAACGTCACACT

#### Western blot assay

Min6 cells were plated in six-well dishes with overnight culture and then incubated with 10 μmol/L tested compounds and 300 μmol/L PA for 12 h. Total proteins of cells were lysed with RIPA lysis buffer (Boster, China) and the protein lysis was separated by SDS-PAGE and transferred from gel to PVDF membrane (Millipore, USA). Then, the membranes were blocked, stained overnight with corresponding primary antibodies and then with second antibody for 1 h. The signals of detected proteins were measured by Chemidoc Imaging System (BIO-RAD, USA). The antibody used were as follows: TXNIP (Abcam, ab188865), NLRP3 (Wanlei, Wuhan, China; WL02635), Cleaved caspase 1 (Wanlei, Wuhan, China; WL03450), Cleaved caspase 3 (CST, #9664s), GAPDH (Bioworld, Nanjing, China; AP0063).

#### Measurement of intracellular reactive oxygen species

Min6 cells were seeded in six-well plates with overnight culture and then incubated with 10 μmol/L tested compounds and 300 μmol/L PA for 8 h. After treatment, cells were cultured with 10 μmol/L DCFH-DA (Meilun Reactive Oxygen Species Assay Kit, China) at 37 °C for 20 min in a humidified atmosphere containing 5% CO_2_. Intracellular ROS levels were visualised using a fluorescence microscope (Olympus IX73, Japan).

### Statistical analysis

All data were expressed as the mean ± standard deviation (SD). Statistical analysis was performed using the GraphPad Prism software, version 8.0 (GraphPad Software Inc., San Diego, CA, USA). A one-way analysis of variance (ANOVA) followed by Tukey’s *post hoc* test was used to evaluate the statistical significance, and *P* < 0.05 was considered statistically significant.

### In silico ADME prediction

Pharmacokinetics (ADME) prediction and drug-likeness properties of the active compounds were analysed using SwissADME, a free available service tool online (http://www.swissadme.ch/).

## Results and discussion

### Chemistry

**Scheme 2.**
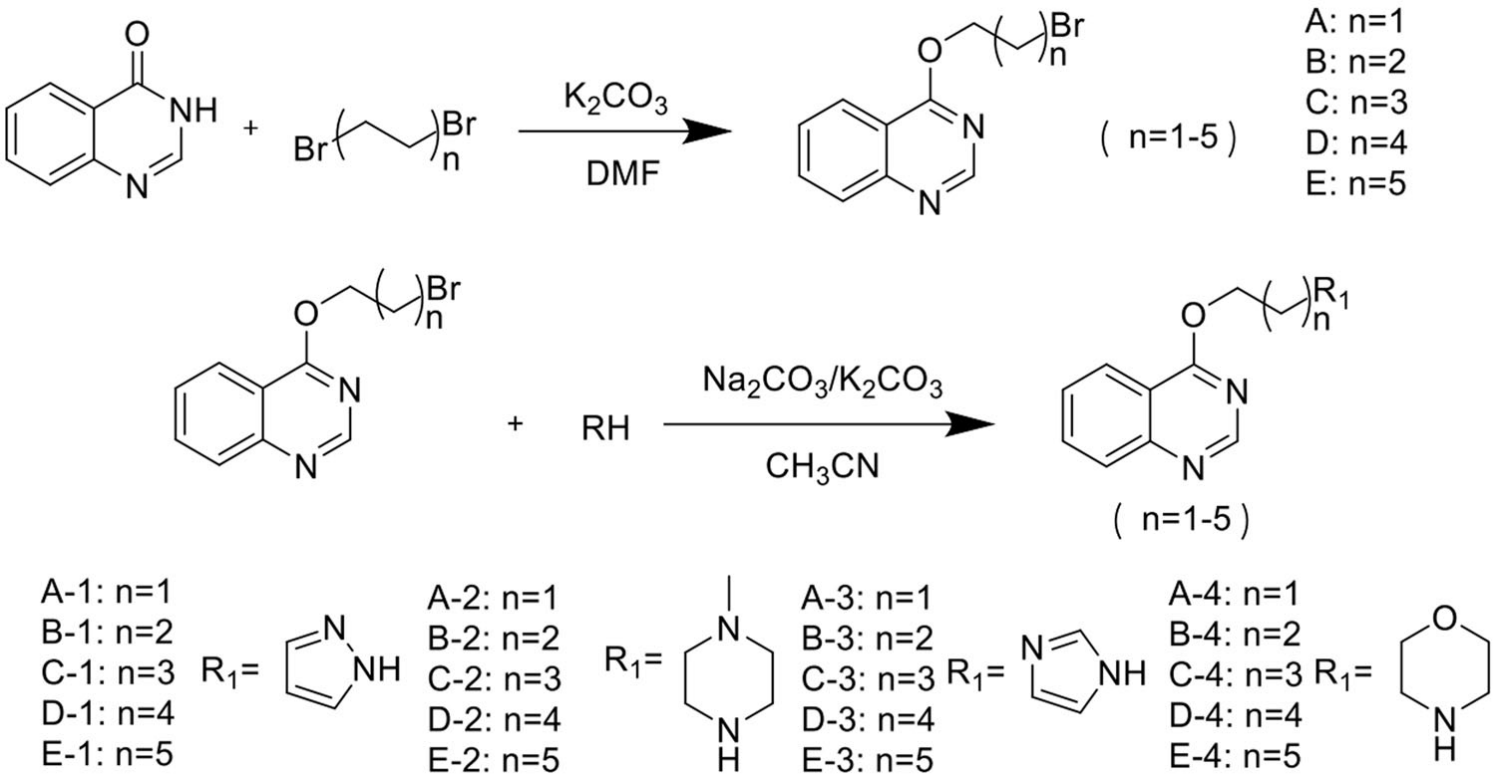
The synthetic route of quinazoline containing nitrogenous heterocyclic derivatives.

The quinazoline derivatives with nitrogen-containing heterocycle contacted by carbon chains of different lengths were synthesised by a two-step reaction (Scheme 2). The first elimination reaction is the removal of H-3 from 4-hydroxyquinazoline in an alkaline environment, which occurs tautomerization, and an oxygen anion is generated. The electron cloud of C-Br is biased towards the bromine atom, which makes α-C have certain electropositivity. Since the bromine atom is an excellent leaving group, the bromine atom is removed to form a C-O bond under the attack of oxygen anion. The second elimination reaction is that the N-H bond in the nitrogen heterocycle has certain acidity. In an alkaline environment, the N-H bond is broken and the N atom is electronegative. The N atom attacks the C-Br bond, causing the bromine atom to leave, forming a new C-N bond.

Chromatography technology was used to purified the 20 compounds which contain different nitrogen-containing heterocycles. Their structures are shown in Table 2. The analytical and spectroscopic data of all compounds were shown as details in the experimental section.

**Table 2.** The structure of 20 quinazoline derivatives.

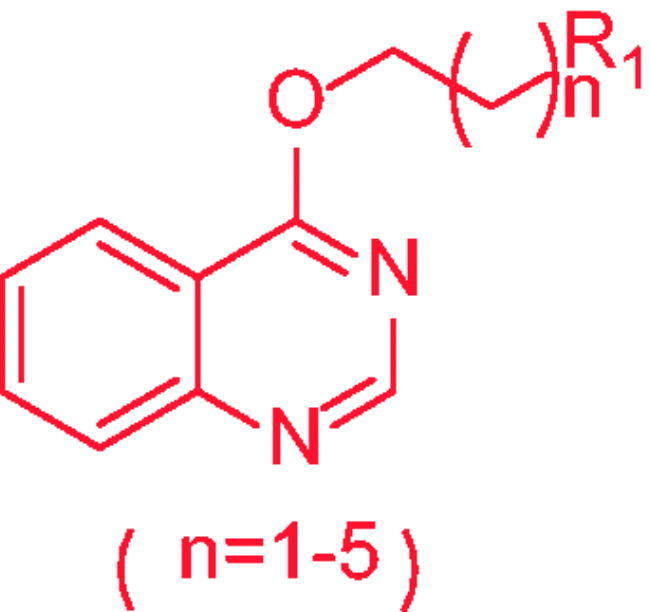					
Compound no.	n	R_1_	Compound no.	n	R_1_
A-1	1	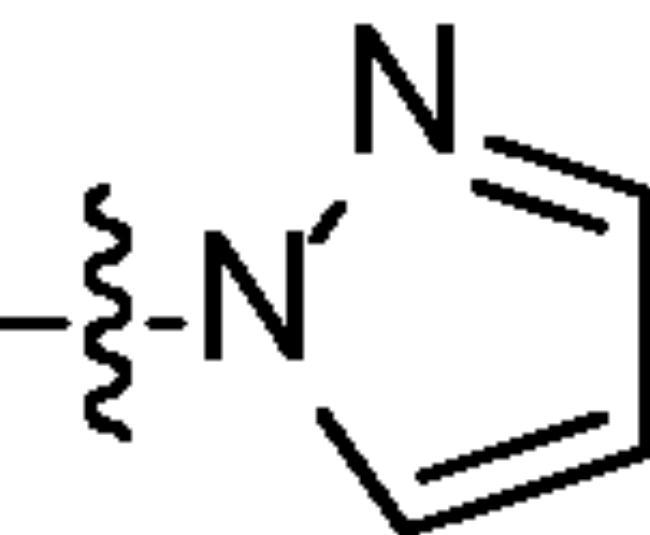	C-3	3	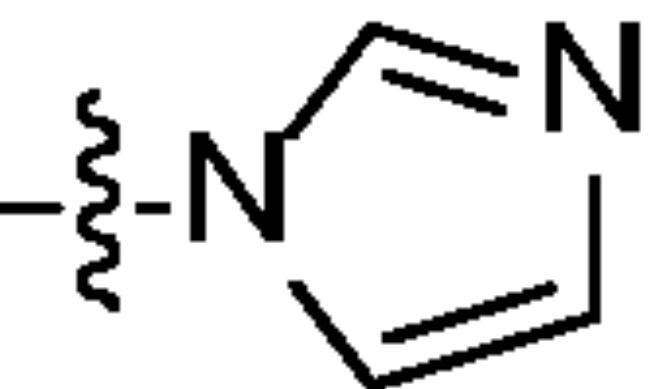
A-2	1	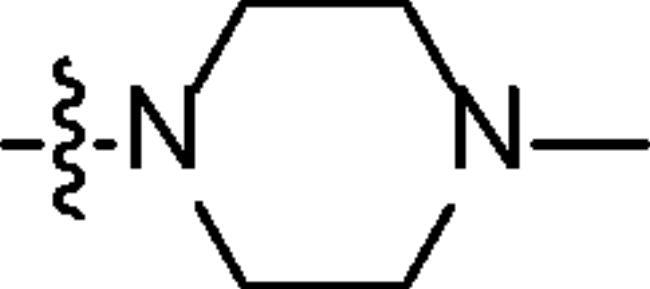	C-4	3	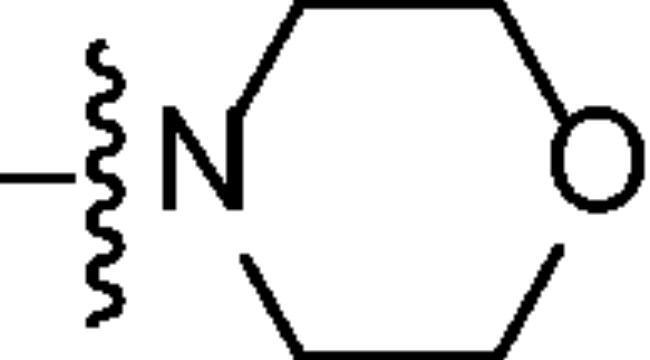
A-3	1	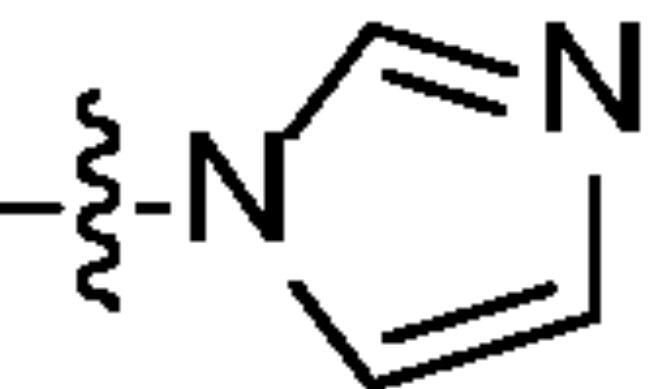	D-1	4	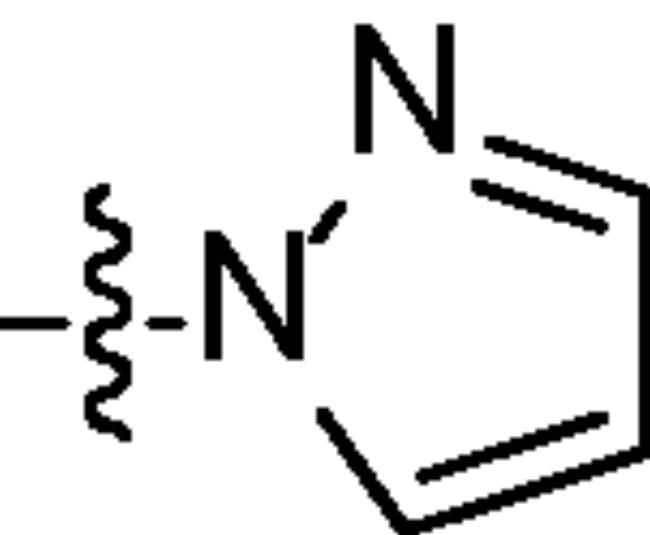
A-4	1	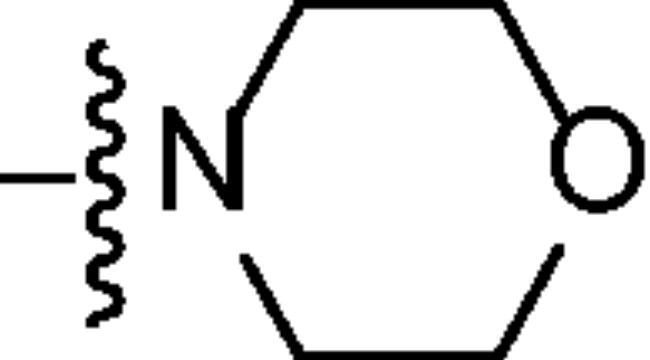	D-2	4	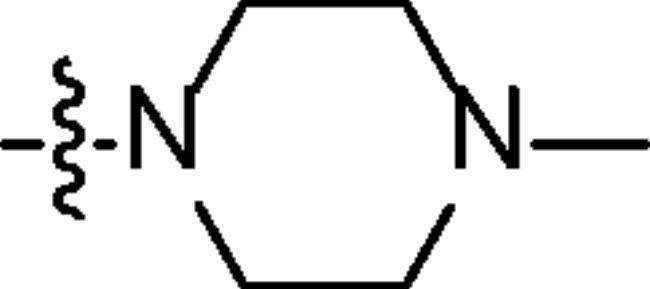
B-1	2	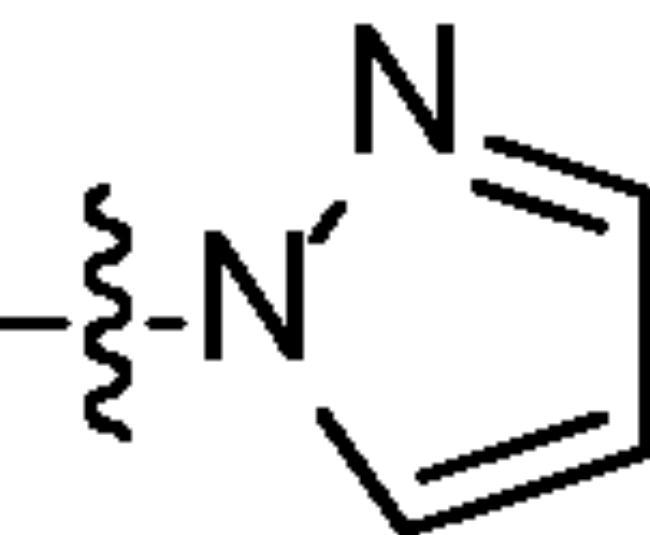	D-3	4	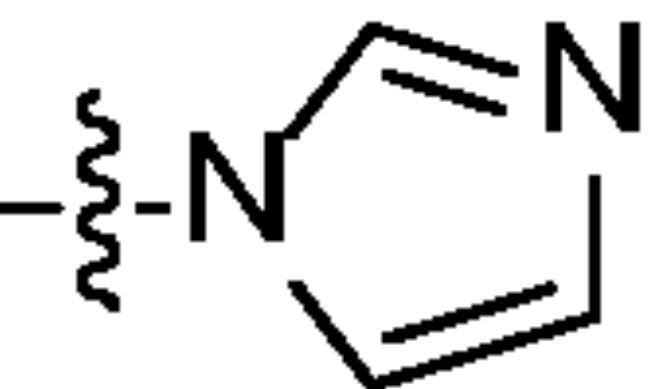
B-2	2	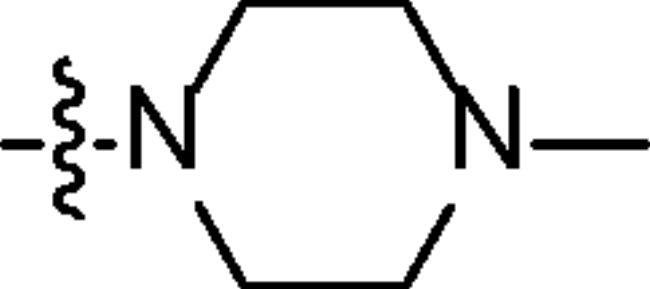	D-4	4	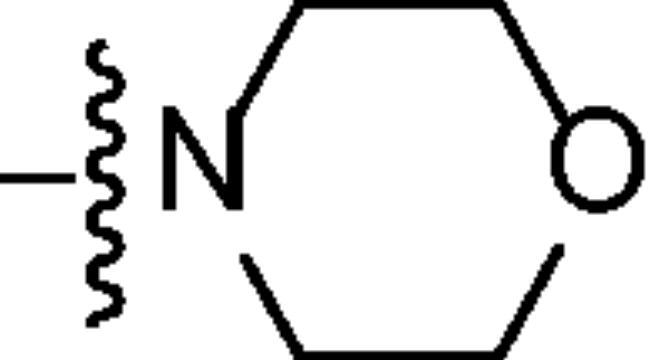
B-3	2	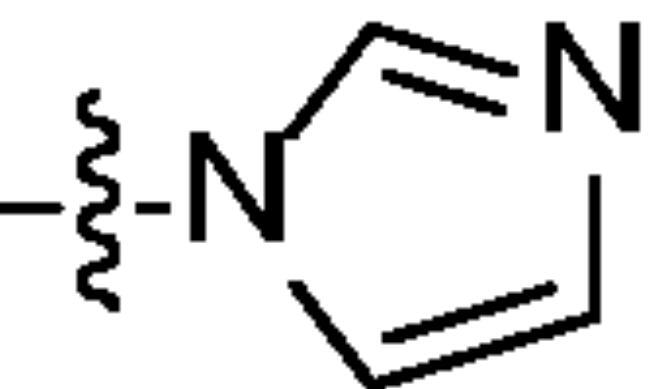	E-1	5	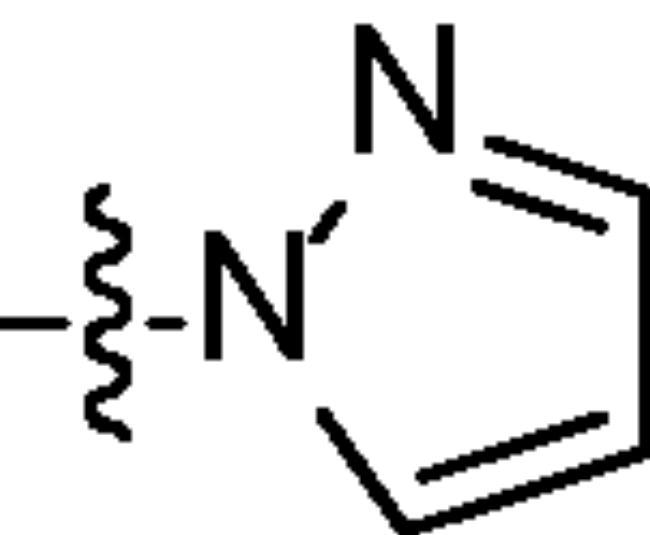
B-4	2	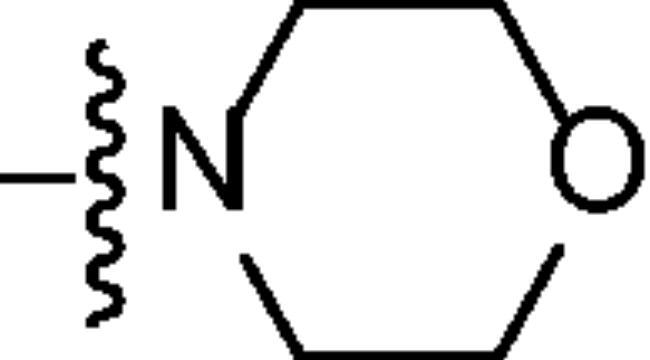	E-2	5	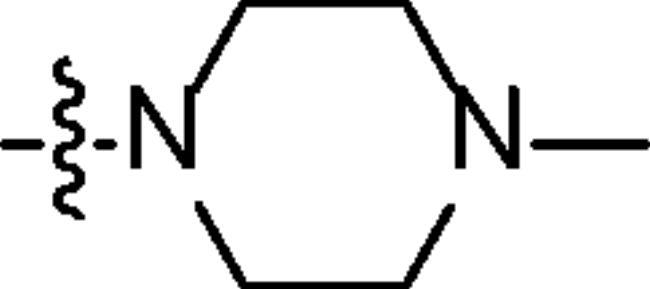
C-1	3	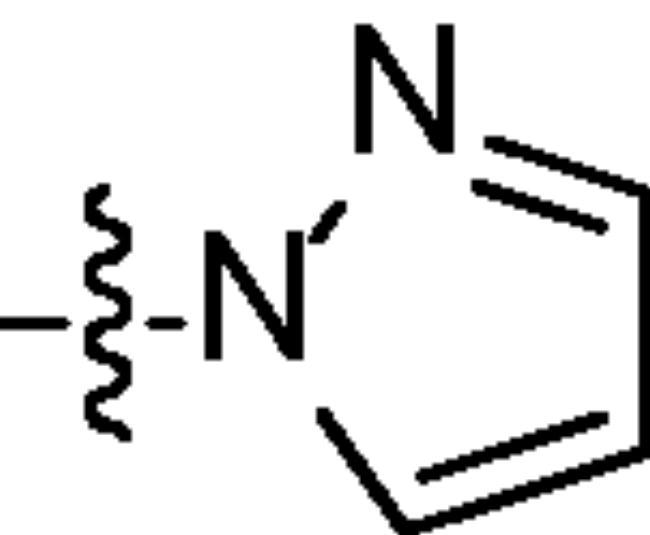	E-3	5	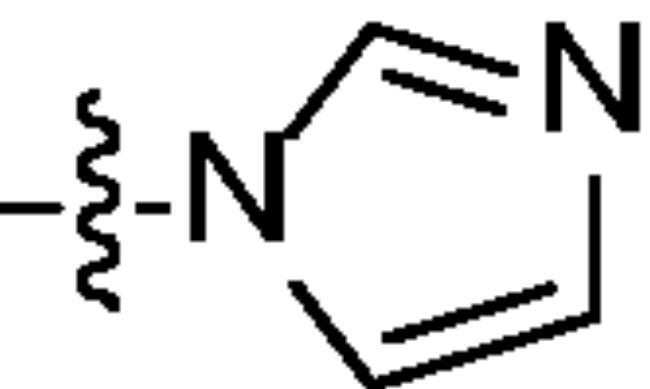
C-2	3	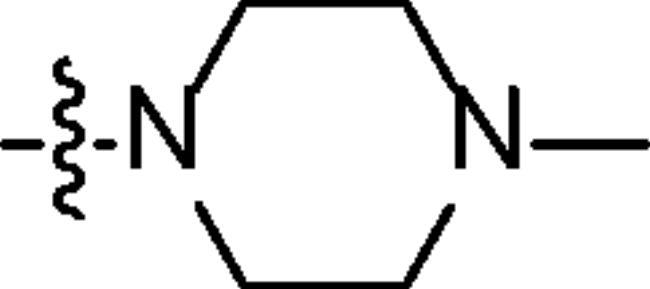	E-4	5	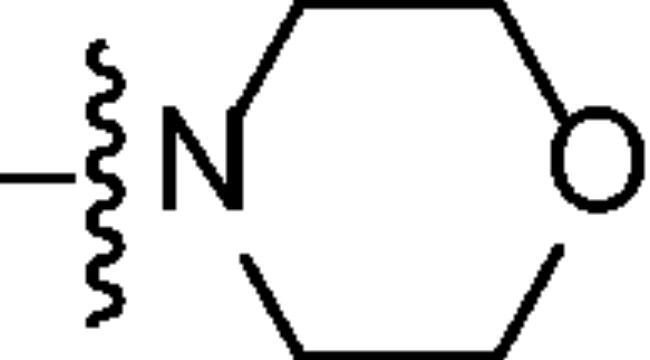

### Protective activity of all compounds in PA-induced Min6 β cell apoptosis

**Figure 1.**
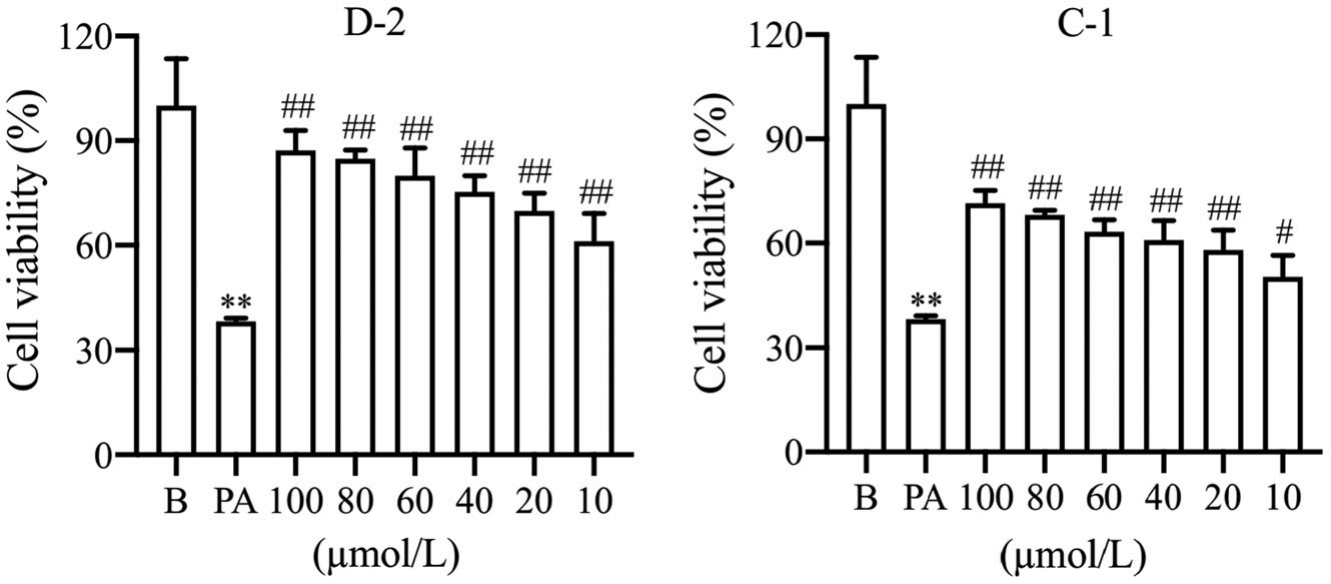
The anti-apoptosis activity with different concentrations of compounds D-2 and C-1. Min6 β cells were incubated with different concentrations of D-2 and C-1 and then exposed to PA (300 μmol/L) for 24 h. **p < 0.01 vs control cells; ^#^*p* < 0.05, ^##^*p* < 0.01 vs PA-treated cells. Data were presented as mean ± SD (*n* = 5).

To investigate the potential protective effect of the target compounds against β cell damage. The anti-apoptosis activity was evaluated in PA-treated Min6 β cell using a CCK-8 assay kit. As shown in Table 3, the cell viability of Min6 β cell treated with PA decreased to 38.3%. However, most of the target compounds, except for A-3, A-4, B-4, and E-1∼E-3, exhibited increased cell viability at a concentration of 100 μmol/L compared to PA treatment. The structure-activity relationship analysis showed that the target compounds could significantly protect β cell when *n* = 3 or 4, and there was no apparent protective activity when *n* = 5 except that R1 was a morpholine ring. Cytoprotective effects were also observed in compounds when the alkane chain lengths were 1 or 2 except that R1 was an imidazole ring or a morpholine ring. We found that compound D-2 showed significant anti-apoptosis activity with 87.2% cell viability, and followed by the compound C-1, 71.5%. So, we chose compounds D-2 and C-1 for the subsequent examinations. Different concentrations of compounds D-2 and C-1 were added in PA-treated β cell and the results showed that compounds D-2 and C-1, even the concentration of 10 μmol/L, could protect PA-induced β cell injury significantly, demonstrating their potential protective effect against β cell damage (Figure 1). Therefore, compounds D-2 and C-1 at concentration of 10 μmol/L were used as representative compounds to investigate their protection against PA-induced islet β cell injury.

**Table 3.** The anti-apoptosis activity of quinazoline derivatives at a concentration of 100 μmol/L.

Comp no.	Cell viability	Comp no.	Cell viability	Comp no.	Cell viability
A-1	64.7%	B-4	28.8%	D-3	62.1%
A-2	58.1%	C-1	71.5%	D-4	62.6%
A-3	36.2%	C-2	70.9%	E-1	32.8%
A-4	27.6%	C-3	40.3%	E-2	26.7%
B-1	67.0%	C-4	71.4%	E-3	26.3%
B-2	45.7%	D-1	42.7%	E-4	70.5%
B-3	39.5%	D-2	87.2%	PA treatment	38.3%

### Compounds D-2 and C-1 inhibited TXNIP expression via promoting protein degradation

**Figure 2.**
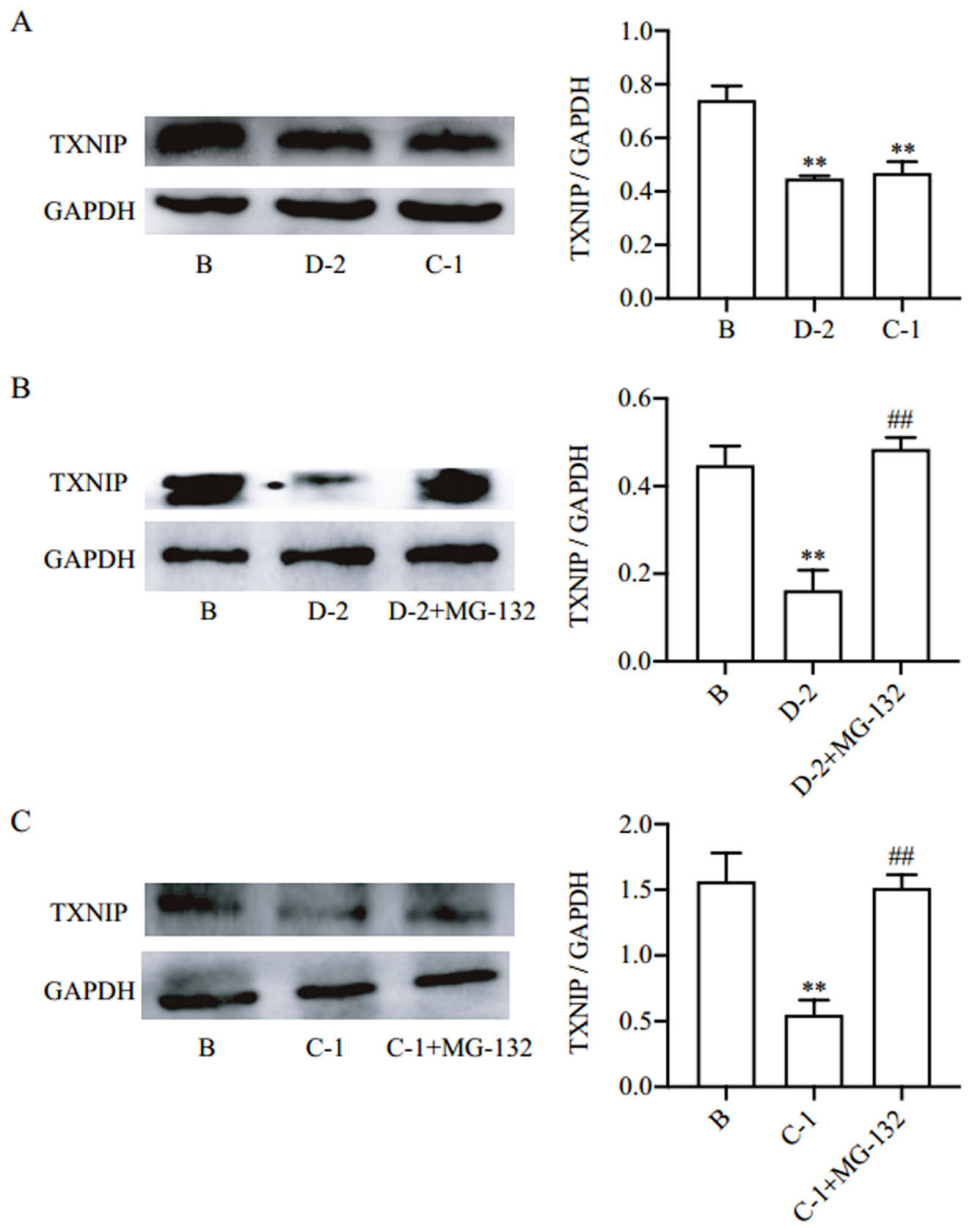
Compounds D-2 and C-1 inhibited TXNIP expression via promoting protein degradation. (A) Min6 β cells were incubated with D-2 and C-1 at concentration of 10 μmol/L for 24 h. (B) Min6 β cells were incubated with cycloheximide (50 μmol/L) and D-2 (10 μmol/L) with or without addition of MG-132 (10 μmol/L) for 24 h. (C) Min6 islet β cells were incubated with cycloheximide (50 μmol/L) and C-1 (10 μmol/L) with or without addition of MG-132 (10 μmol/L) for 24 h. Protein expression of TXNIP was measured by Western blot. **p < 0.01 vs control cells; ^##^*p* < 0.01 vs indicated cells. Data were presented as mean ± SD (*n* = 4).

Then, the inhibitory effect of compounds D-2 and C-1 on TXNIP expression were examined. In physiological conditions, compounds D-2 and C-1 could inhibit TXNIP protein expression in Min6 β cell (Figure 2(A)). In fact, protein expression levels depend on the amount of mRNA for synthesis or the rate of ubiquitination for degradation, the balance between irreversible processes synthesis and degradation^24^. To confirm the underline mechanism by which these two compounds inhibit TXNIP protein expression. Cycloheximide was added in Min6 β cells to inhibit protein synthesis, and compounds D-2 and C-1 could decrease TXNIP protein expression, while these actions were abrogated by proteasome inhibitor MG-132. Based on the design concept of TXNIP inhibition, the results indicated that compounds D-2 and C-1 inhibited TXNIP protein expression via promoting protein degradation (Figure 2(B,C)).

### Cellular mechanism of action by compounds D-2 and C-1

Next, the underline mechanism by which TXNIP inhibition by compounds D-2 and C-1 could protect Min6 β cells from PA insult. Given that inhibition of TXNIP could reduce oxidative stress and inflammatory injury of pancreatic β cell in T2DM^20^^,^^22^, effects of compound D-2 and C-1 on ROS production and inflammatory signalling in the context of PA insults were assayed.

#### Compound D-2 and C-1 reduced intracellular ROS generation in PA-induced Min6 cells

**Figure 3.**
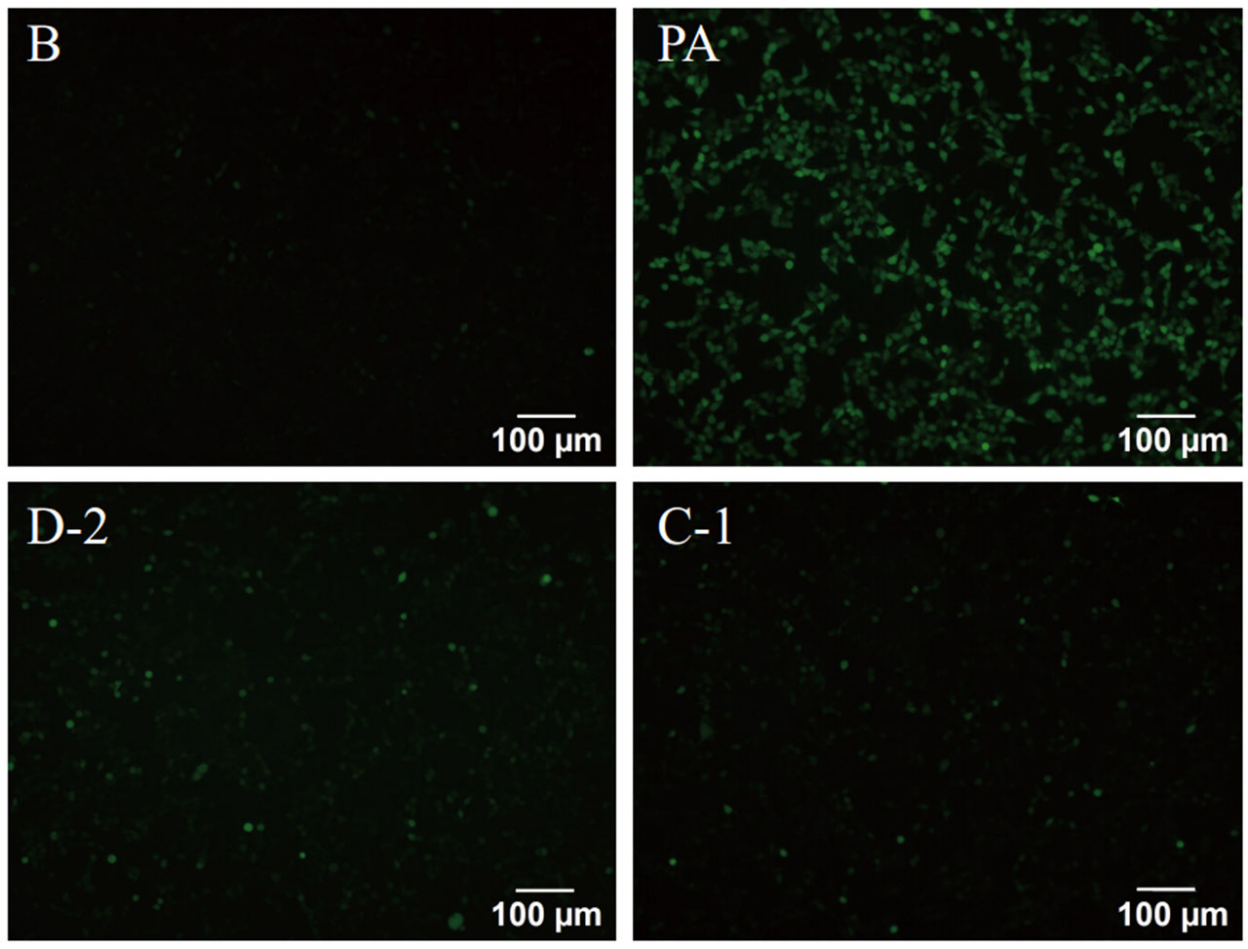
Compounds D-2 and C-1 reduced ROS generation in PA-induced Min6 cells. Min6 β cell were incubated with D-2 and C-1 at concentration of 10 μmol/L and then exposed to PA (300 μmol/L) for 8 h. Immunofluorescence staining for ROS was photographed by fluorescence microscopy. Bar: 100 μm.

Reactive oxygen species (ROS) are essential signalling molecules that regulate physiological cell functions^25^. However, overproduction of ROS by excess FFAs results in oxidative stress and cell death^26^^,^^27^. ROS can not only directly damage cells or tissues but also indirectly activate a series of damage-related signalling pathways. Given that peroxisomal FFAs β-oxidation generates H_2_O_2_ and β cell lack the H_2_O_2_-inactivating enzyme catalase in peroxisomes, β cell are highly susceptible to oxidative stress compared to other cell types^28^. Hence, intracellular ROS generation was detected by fluorescence assay. The result showed that PA treatment obviously increased intracellular ROS production, while compounds D-2 and C-1 could inhibit ROS generation and significantly reduce intracellular ROS levels, and thus protecting β cell from PA-induced oxidative stress damage (Figure 3).

#### Compounds D-2 and C-1 protected Min6 cells from PA-induced inflammatory injury via TXNIP-NLRP3 signalling

**Figure 4.**
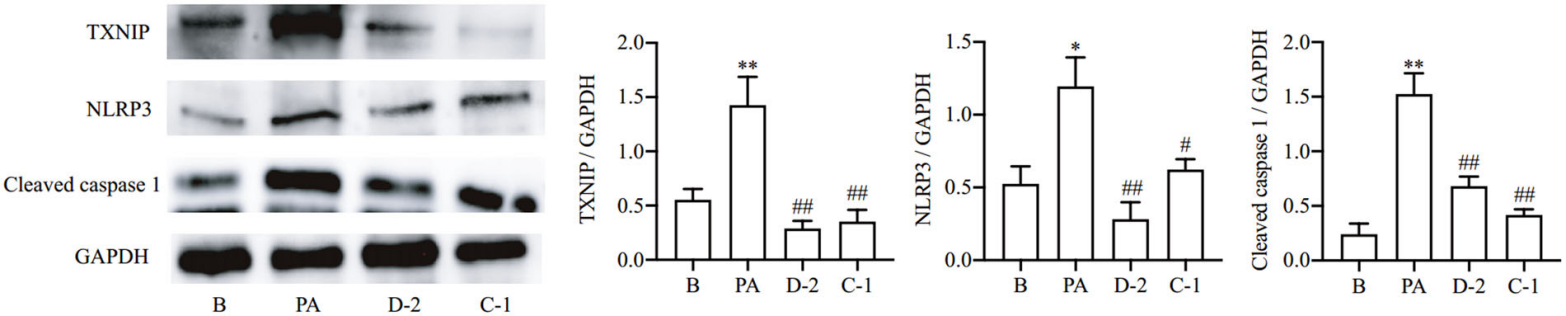
Compounds D-2 and C-1 protected Min6 cells from PA-induced inflammatory injury via TXNIP-NLRP3 signalling. Min6 β cell were incubated with D-2 and C-1 at concentration of 10 μmol/L and then exposed to PA (300 μmol/L) for 12 h. Protein expressions of TXNIP, NLRP3 and cleaved caspase 1 were measured by Western blot. *p < 0.05 & **p < 0.01 vs control cells; ^#^*p* < 0.05 & ^##^*p* < 0.01 vs PA-treated cells. Data were presented as mean ± SD (*n* = 4).

As a critical factor in cellular redox balance, TXNIP is a bridge between oxidative stress and inflammatory injury via interaction with NLRP3 inflammasome^20^^,^^29^. The NLRP3 inflammasome is composed of NLRP3, recruitment domain (ASC), and pro-caspase-1, and its activation cleaves pro-caspase-1 into its active forms and leads to IL-1β maturation and release^30^^,^^31^. This cascade triggers sustained inflammation, which has been implicated in metabolic syndrome and diabetes^32^. As shown in Figure 4, TXNIP, NLRP3 and cleaved caspase 1 protein expressions were significantly induced in Min6 β cell exposure to PA. However, these alterations were reversed by culturing with compounds D-2 and C-1, demonstrating that compounds D-2 and C-1 could protect Min6 cells from PA-induced inflammatory injury via inhibiting TXNIP-NLRP3 signalling.

#### Compounds D-2 and C-1 inhibited gene expressions of inflammatory cytokines

**Figure 5.**
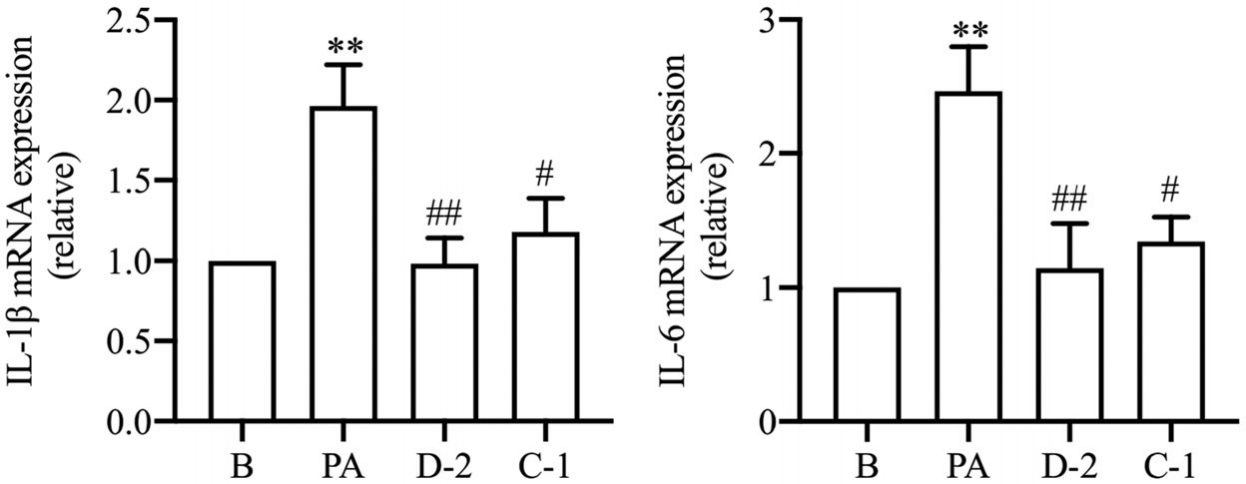
Compounds D-2 and C-1 inhibited gene expression of inflammatory cytokines. Min6 β cell were incubated with D-2 and C-1 at concentration of 10 μmol/L and then treated with PA (300 μmol/L) for 24 h. Gene expressions of IL-1β and IL-6 were detected using the RT-PCR method. **p < 0.01 vs control cells; ^#^*p* < 0.05 & ^##^*p* < 0.01 vs PA-treated cells. Data were presented as mean ± SD (*n* = 5).

Saturated FFAs can trigger inflammatory pathways in β cell via direct activation or secondarily to oxidative and ER stress^33^. PA induces NLRP3 inflammasome activation, and the expression/maturation of cytokines, such as IL-1β and IL-6 could potentially induce β cell dysfunction or apoptosis and has been implicated in the pathogenesis of T2DM^34^. As shown in Figure 5, PA treatment significantly increased IL-1β and IL-6 gene expressions in Min6 cells. Compounds D-2 and C-1 decreased IL-1β and IL-6 gene expressions in Min6 cells exposed to PA, showing their anti-inflammatory activity. Macrophage levels are elevated in the pancreatic islets of T2DM and macrophages drive β cell failure and apoptosis, resulting in chronic low-grade inflammation in the pancreatic islets^35^.

#### Compounds D-2 and C-1 inhibited caspase 3 activation

**Figure 6.**
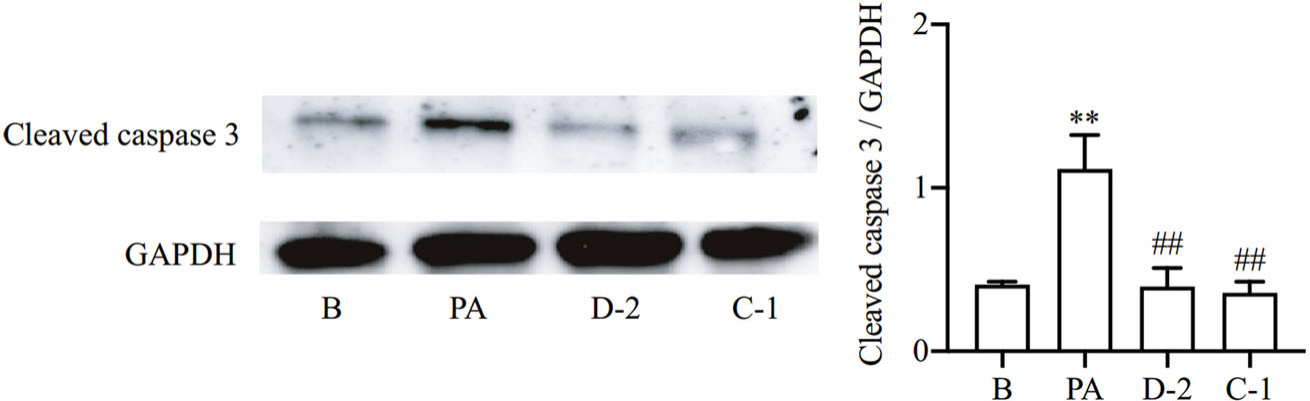
Compounds D-2 and C-1 inhibited caspase 3 activation. Min6 β cells were incubated with D-2 and C-1 at concentration of 10 μmol/L and then exposed to PA (300 μmol/L) for 12 h. Protein expression of cleaved caspase 3 was measured by Western blot. **p < 0.01 vs control cells; ^##^*p* < 0.01 vs PA-treated cells. Data were presented as mean ± SD (*n* = 4).

Oxidative stress and inflammation are major cause for β cell apoptosis. Cysteinyl aspartate specific proteinase (caspase) family has been proven to be involved in β cell apoptosis. Compared with other members of the caspase family, caspase 3 is at the end of the caspase cascade and exists as the terminal executor of cell apoptosis^36^. Caspase-3 is an inactive proenzyme that can be cleaved by initiator caspases to induce the activation of caspase-3 during apoptotic signalling. Therefore, the protein expression of cleaved caspase 3 was measured by Western blot to evaluate the protective effect of compounds D-2 and C-1 in β cell when exposed to PA. The results showed that PA treatment significantly increased cleaved caspase 3 expression, while compounds D-2 and C-1 inhibited caspase 3 activation in PA-treated Min6 β cell, demonstrating that compound D-2 and C-1 could inhibit PA-induced β cell apoptosis (Figure 6).

### In silico drug-likeness and ADME predictions of compounds D-2 and C-1

**Figure 7.**
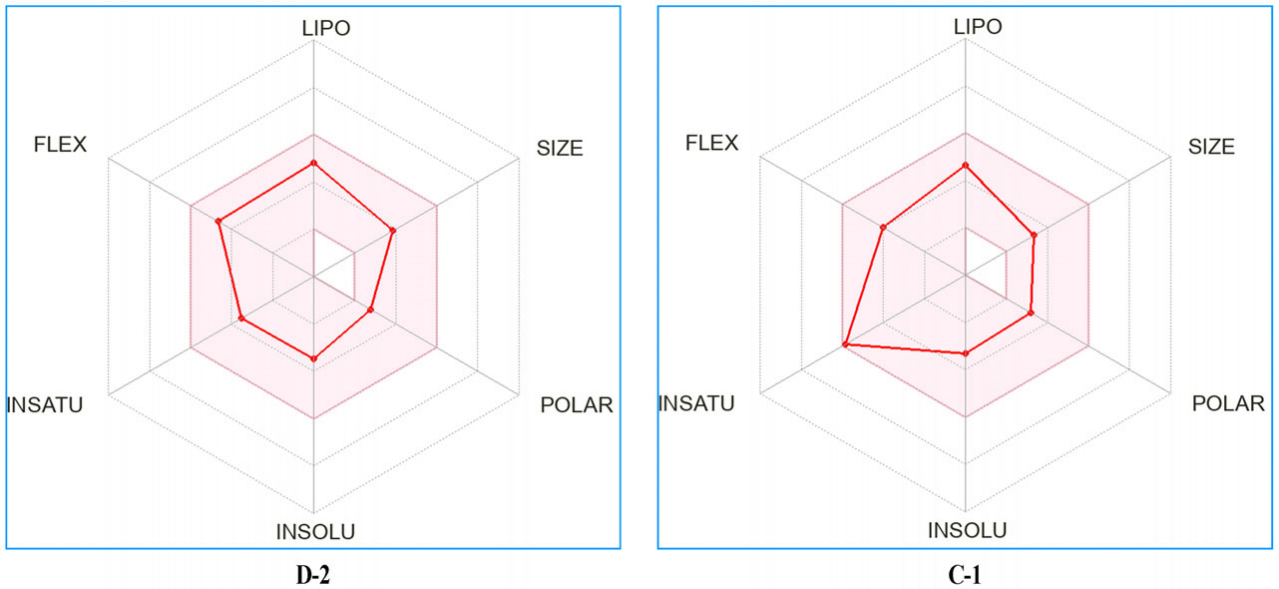
Bioavailability radar plot of compounds D-2 and C-1.

Given that efficacy and safety are vital aspects for a potential drug molecule in the process of drug development, we predicted physicochemical and pharmacokinetic parameters of the compounds D-2 and C-1 with a free online available website SwissADME^37^. The predicted physicochemical properties of the compounds D-2 and C-1 were depicted in the bioavailability radar plot (Figure 7). Bioavailability radar plot represents six physicochemical properties (See 1–6 in Table 4) to reflect oral bioavailability, and is displayed for a rapid appraisal of drug-likeness^37^. Compounds with radar plot (red line) entirely fallen into the pink area are considered optimal drug-likeness. According to the Figure 7, compound D-2 and C-1, particularly the compound D-2, process comparable drug-likeness.

**Table 4.** Physicochemical and pharmacokinetic parameters of compounds D-2 and C-1 predicted with SwissADME online tool.

Parameters	Compound D-2	Compound C-1
1. Size, molecular weight	314.43	268.31
2. Saturation, fraction Csp3	0.56	0.27
3. Polarity, topological polar surface area (Å^2^)	41.49	52.83
4. Lipophilicity, Log P_o/w_	2.66	2.48
5. Solubility, Log S	−3.47	−3.31
6. Flexibility, num. rotatable bonds	7	6
7. Num. H-bond acceptors	5	4
8. Num. H-bond donors	0	0
9. GI absorption	High	High
10. BBB permeant	Yes	Yes
11. Drug-likeness	Yes	Yes

The predicted pharmacokinetic parameters showed that compound D-2 and C-1 were highly permeable to gastrointestinal (GI) membranes and could pass through the blood-brain barrier (BBB) (See 9–10 in Table 4). Besides, other pharmacokinetic properties such as substrate or non-substrate of the permeability glycoprotein (P-gp), whether interaction with the five major cytochromes P450 (CYP) isoforms, and skin permeation were also predicted by SwissADME (Supplementary Figure 1 and 2). In addition, compound D-2 and C-1 satisfy the five drug-likeness rules and showed medicinal chemistry leadlikeness (Supplementary Figures 1 and 2).

## Conclusions

In summary, a series of quinazoline derivatives were designed, synthesised, and evaluated to inhibit TXNIP expression and protect PA-induced β cell injury for T2DM treatment. Most of these compounds could protect β cell from PA insult and compound D-2, as star molecule, along with compound C-1 could effectively promote β cell survival and the viability of β cell reached 61.1% and 50.3% at concentration of 10 μmol/L, respectively, compared to the β cell injury model 38.3%. Subsequent results showed that compounds D-2 and C-1 inhibited TXNIP expression by promoting its protein degradation. Besides, the cellular mechanism of action studies demonstrated that compounds D-2 and C-1 could reduce the production of ROS and inhibit the TXNIP-NLRP3 inflammasome signalling pathway under PA stimulation, and thus alleviating oxidative stress and inflammatory response, thereby reducing caspase 3 activation and protecting PA-induced Min6 β cell damage. Furthermore, according to their predicted drug-likeness and medicinal chemistry properties, both compounds, particularly the compound D-2, could be better lead drug candidates. Overall, the present study evidently showed that quinazoline nitrogen-containing heterocyclic derivatives to inhibit TXNIP would be potent pancreatic β cell protective agents and compounds D-2 and C-1, especially the compound D-2, might serve as promising lead candidates for the treatment of T2DM.

## Supplementary Material

Supplemental Material
