# Supplementary material for: TXNIP inhibition in the treatment of type 2 diabetes mellitus: design, synthesis, and biological evaluation of quinazoline derivatives
Source: J Enzyme Inhib Med Chem. 2023 Jan 18;38(1):2166937. doi: 10.1080/14756366.2023.2166937 (PMC9858527; doi:10.1080/14756366.2023.2166937)
Supplement: Supplemental Material [file IENZ_A_2166937_SM4907.pdf]

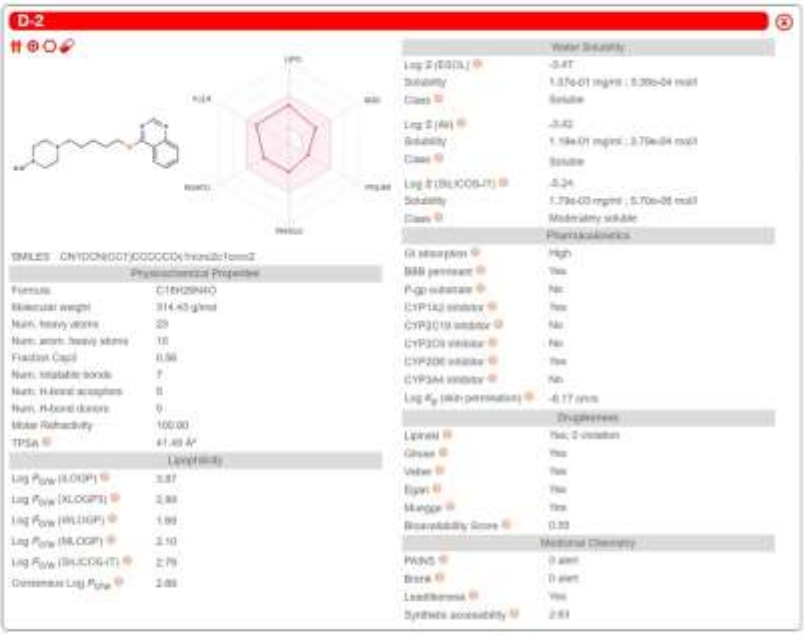

Supplementary Figure 1. Physicochemical and pharmacokinetics properties of compound D-2 predicted using SwissADME.

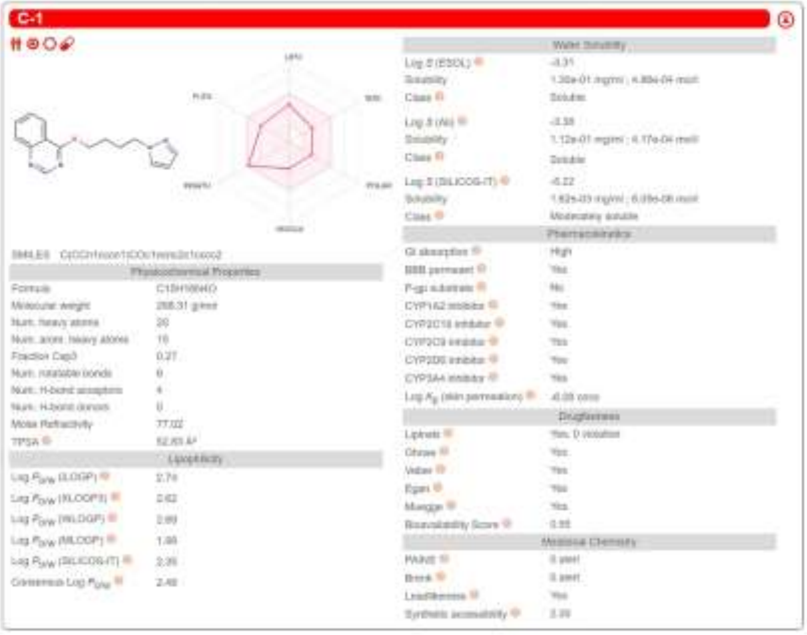

Supplementary Figure 2. Physicochemical and pharmacokinetics properties of compound C-1 predicted using SwissADME.

Information Classification: General
